# Resliced image space construction for coronary artery collagen fibers

**DOI:** 10.1371/journal.pone.0184972

**Published:** 2017-09-27

**Authors:** Tong Luo, Huan Chen, Ghassan S. Kassab

**Affiliations:** California Medical Innovations Institute, San Diego, California, United States of America; Pennsylvania State Hershey College of Medicine, UNITED STATES

## Abstract

Collagen fibers play an important role in the biomechanics of the blood vessel wall. The objective of this study was to determine the 3D microstructure of collagen fibers in the media and adventitia of coronary arteries. We present a novel optimal angle consistence algorithm to reform image slices in the visualization and analysis of 3D collagen images. 3D geometry was reconstructed from resliced image space where the 3D skeleton was extracted as the primary feature for accurate reconstruction of geometrical parameters. Collagen fibers (range 80–200) were reconstructed from the porcine coronary artery wall for the measurement of various morphological parameters. Collagen waviness and diameters were 1.37 ± 0.19 and 2.61 ± 0.89 μm, respectively. The biaxial distributions of orientation had two different peaks at 110.7 ± 25.2° and 18.4 ± 19.3°. Results for width, waviness, and orientation were found to be in good agreement with manual measurements. In addition to accurately measuring 2D features more efficiently than the manual approach, the present method produced 3D features that could not be measured in the 2D manual approach. These additional parameters included the tilt angle (5.10 ± 2.95°) and cross-sectional area (CSA; 5.98 ± 3.79 μm^2^) of collagen fibers. These 3D collagen reconstructions provide accurate and reliable microstructure for biomechanical modeling of vessel wall mechanics.

## Introduction

The mechanical properties of blood vessels largely stem from the microstructure, which varies in different organs and tissues. A structure-specific mechanical model is essential not only for better understanding the biomechanics of blood vessels, but also necessary for clarifying the initiation, progression, and clinical treatment of vascular diseases [1–3]. The earliest microstructural model was proposed by Lanir 40 years ago, followed by a number of micromechanical models in the ensuing decades [4]. These models differ mainly with respect to the structure pattern of main components: number of tissue layers, scale, liquid or solid nature of ground substance, smooth muscle cells (SMC), fiber orientation, and undulation of fibers. The arterial wall consists of three major microstructural components: collagen, elastin, and SMC. Collagen is considered to play the most important role in arterial wall strength and load bearing. Realistic data for use in modeling depends on geometrical reconstruction by 3D image processing.

From an image processing perspective, the 3D collagen structure with undulation and tortuosity is the most challenging for segmentation. Tracking by Vaa3D, NeuTu and other toolboxes [5–7] requires relatively high image quality and certain structure assumptions, which are not feasible for all collagen fiber images. In our previous study, 3D elastin fiber geometry was reconstructed [8]. Here, the aim is to reconstruct 3D geometry of collagen fibers in the vascular wall to provide full integration of the adventitia and media models. By incorporating adventitia structure into media, the results of this study will lead to better understanding of the entire vessel wall microstructure required for biomechanical modeling.

Multi-Planar Reconstruction (MPR) or Curved Planar Reformation (CPR) is a typical re-sampling technique to depict vessel cross section structure more clearly where curve- or skeleton-driven slicers have been investigated [9, 10]. Limitations of CPR are operator-dependence and artifact introduction. Direct implementation of CPR in collagen fiber analysis is not practical, as the fiber skeleton is difficult to extract when the structure cannot be clearly delineated. In this study, the novel reslicing strategy focused on search of optimal angle difference when construction of the curved planar reformation which has not been previously done. The arbitrary planar concept was analyzed using new consistency implementation. The tortuousity structure extraction was converted into easier region segmentation.

## Methods

### Sample preparation

The hearts of healthy pigs (n = 7, body weight in the range of 80–90 kg) were obtained from a local slaughterhouse. Hearts were transported to the laboratory in 4°C physiological solution (0.9% NaCl) within 1 hr. after the animals were sacrificed. The left anterior descending (LAD) coronary artery was dissected carefully from its emergence at the aortic ostia and removed with adjacent connective tissue. The details of data collection on collagen fibers from the adventitia and media can be found in previous publications [11, 12]. Briefly, unstained fresh adventitia specimens of LAD arteries were imaged under varied mechanical loads.

### Data imaging

**Fig 1:**
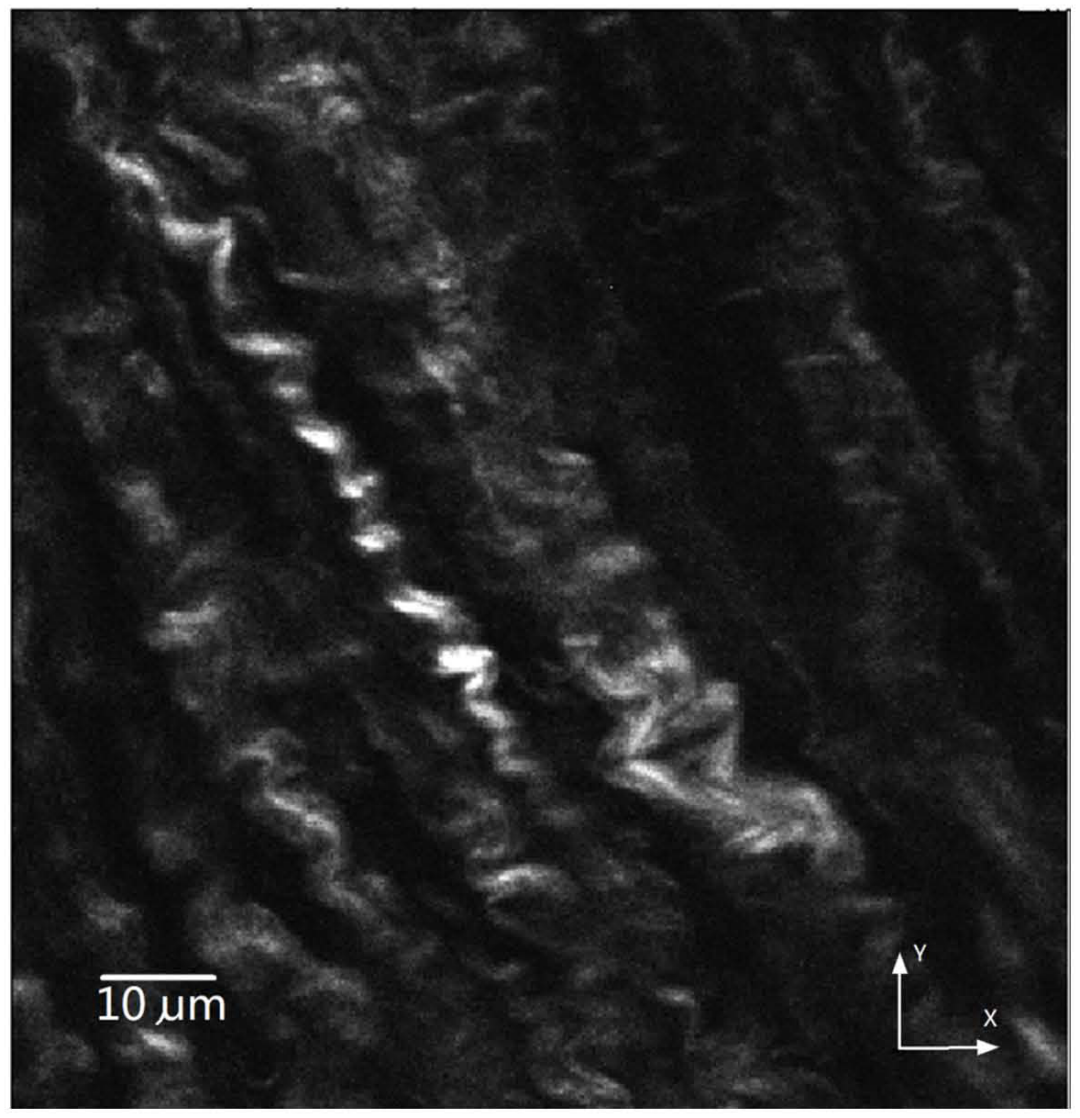
Original images of collagen fibers.

**Fig 2:**
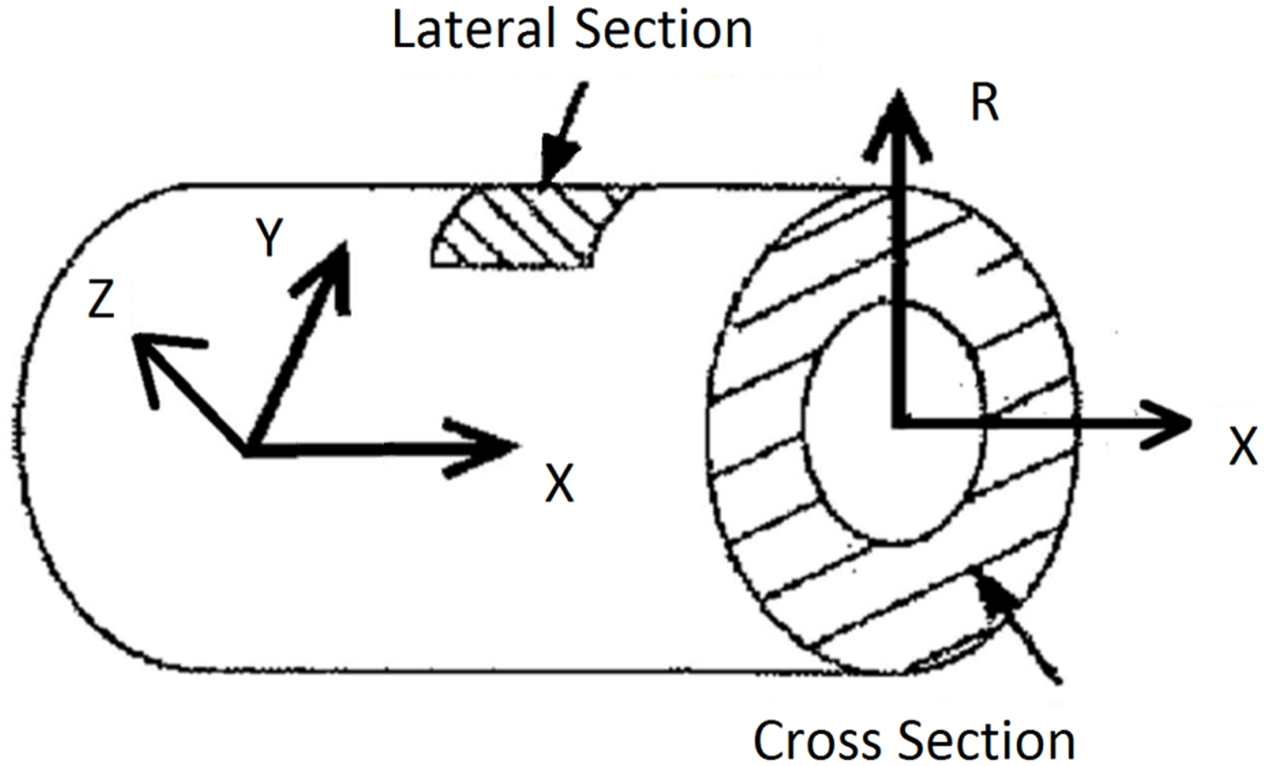
Coordinate system used to define the vessel surface.

Samples were scanned with a combined multiphoton microscopy (MPM) system capable of collecting both second harmonic generation (SHG) and two-photon excited fluorescence (TPEF) signals. TPEF is used to depict elastin that contains endogenous fluorophores, whereas the SHG signal originates from collagen type I that contains molecular noncentrosymmetric structures. The system consists of a mode-locked Ti: Sapphire laser (Chameleon Vision; Coherent Santa Clara, CA) and a 60x1.1NA water immersion objective (Olympus America, Center Valley, PA). The average excitation power at the sample was ~40mWwith an excitation wavelength of 830 nm. The TPEF and backward SHG signals were collected by the same objective and directed toward two external photomultiplier-tube (PMT) detectors. The dichroic mirror (750dcxr; Chroma Technology, Brattleboro, VT) was used to separate detected signals from the excitation beam. For simultaneous imaging, the SHG signal from collagen at 415 nm and TPEF signal from elastin at ~520 nm were separated from each other and directed toward two external PMTs by dichroic mirror (425dcxr; Chroma). The corresponding bandpass filters (405/40 and 520/40 from Chroma) was used in front of PMTs to selectively detect SHG and TPEF signals. Lateral resolution of the system is about 0.25μm. The step size between slices was typically set at 0.25 μm, and the number of slices for one site was limited to 160μm due to the penetration depth. The field of view was 120 x 120 μm^2^, and each acquired image was integrated over two frames to improve the signal-to-noise ratio. A high gain results in a noisy image that requires a greater number of frames to average, thus a low scan speed was used if the gain was too high. An original image for a collagen fiber is demonstrated in Fig 1, while the definition of XYZ coordinates in image stacks is depicted in Fig 2. A total of 80 collagen fibers were analyzed with over 100,000 measurement points of CSA and diameter. Over 200 fibers were used in measurements of tilt and waviness.

For analysis, an arbitrary plane was constructed in simple and straightforward way. Important principles were imposed as follows: 1) Mathematical description of plane; 2) Consistent coordinate axes of created planes; 3) Flexible coordinate definition in a plane; and 4) Conversion of resliced points into MPR/CPR plane.

### Description of plane

**Fig 3:**
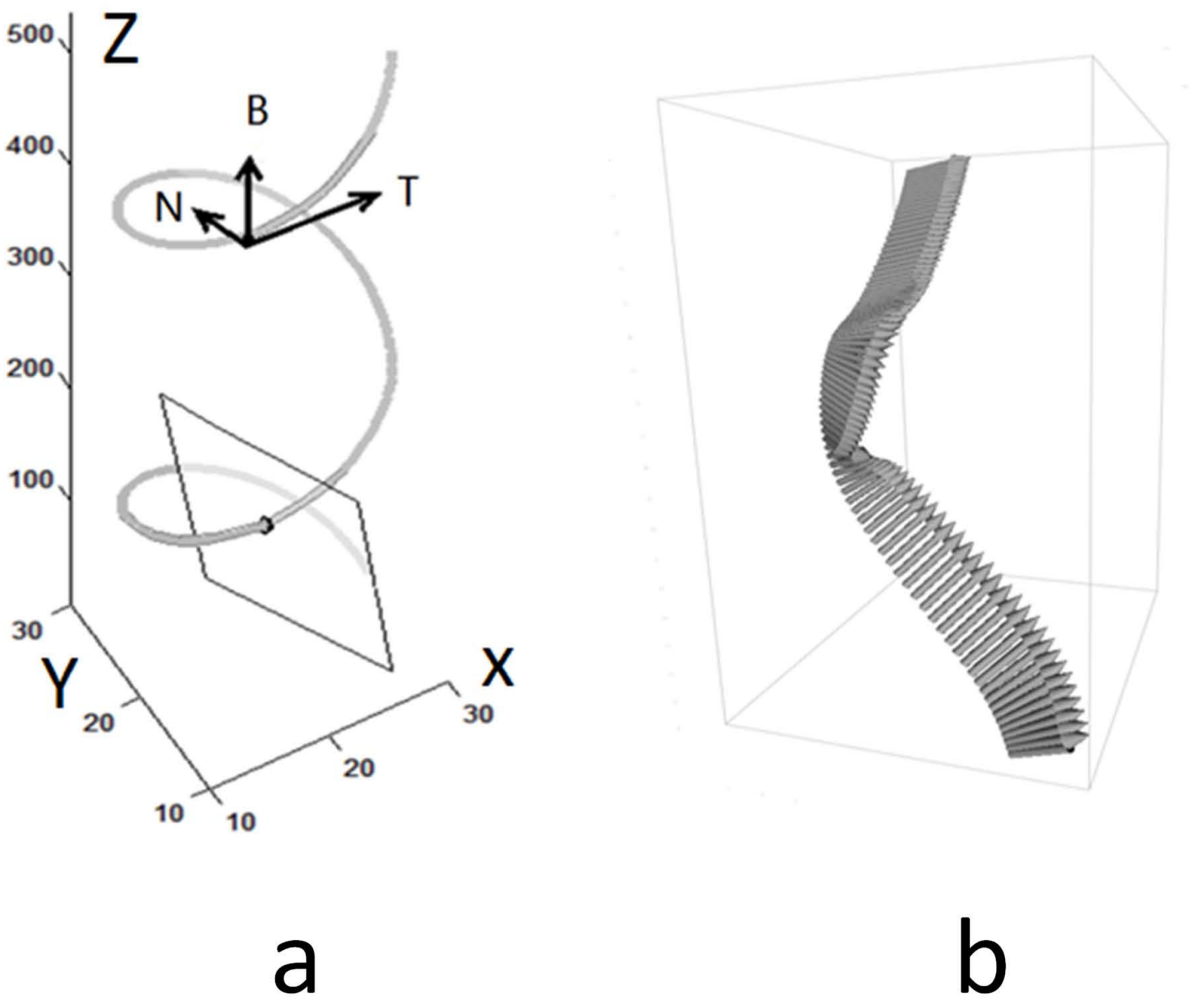
a) Frenet-Serret frame. Vectors N, B, and T denote normal, binormal, and tangent. b) Example of normal vectors along a curve.

**Fig 4:**
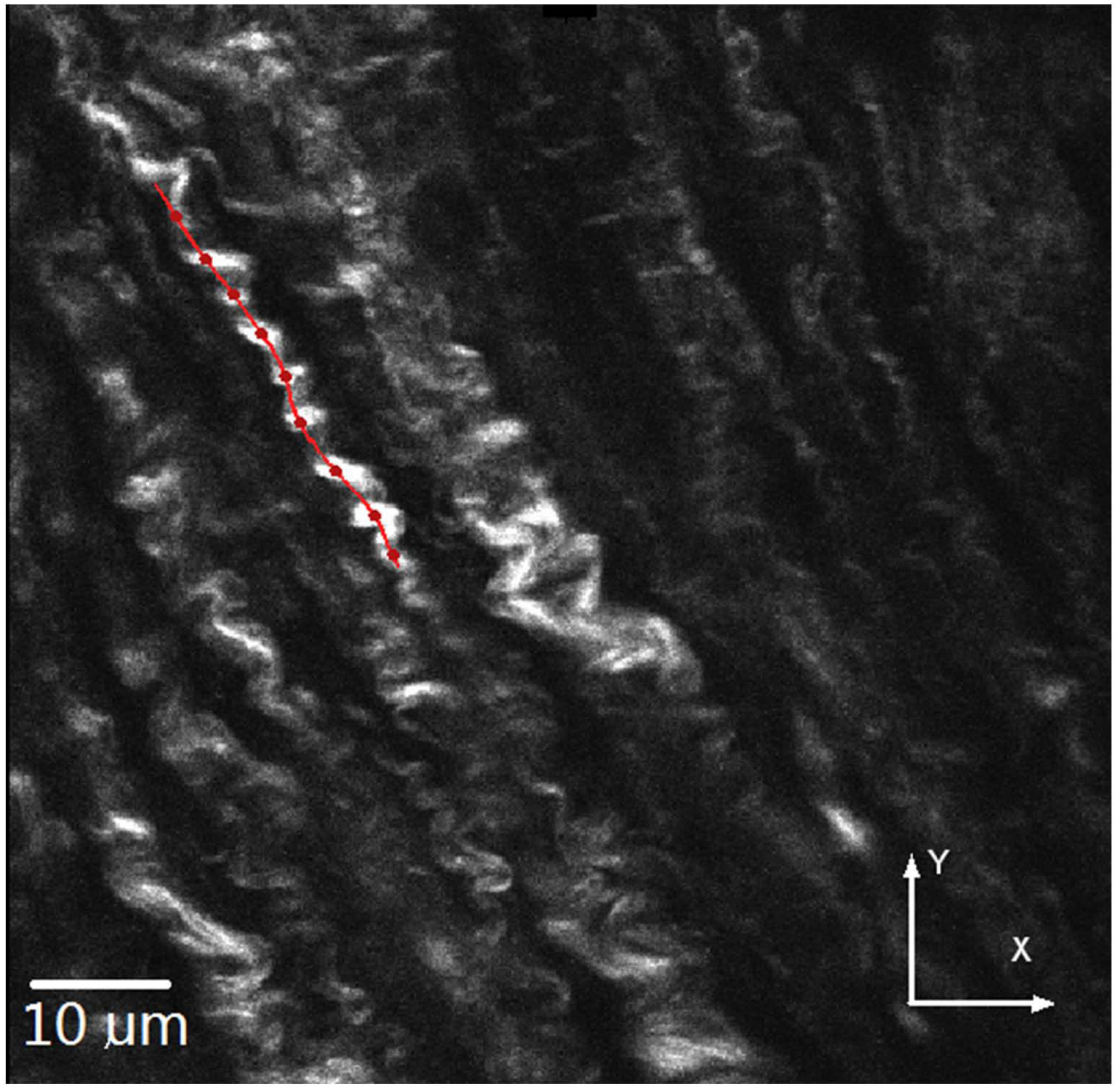
Example of initial curve on collagen image slice.

The fiber skeleton is a 3D curve where the resliced plane is defined as the normal or cross sectional plane along the curve at each point. The relationship between the skeleton and cross section plane is illustrated in Fig 3a. Local axes at curve points were defined as normal, binormal, and tangent under the Frenet-Serret frame. Hence, the curve tangent at each point was defined as *t*_*pi*_, and the resliced plane normal was denoted as: *n*_*pj*_, *n*_*pi*_ = *t*_*pi*_, 1 ≤ *i* ≤ *N*, *i* = *j*. Fig 3b shows the normal vectors are consistent along curve points in a perspective view. The initial skeleton in Fig 4 was manually drawn or detected by CT-FIRE or SOAX method.

### Consistent coordinate axes

When the signs of curvature change, there could can be a null normal (i.e., no torsion of the curve and correct plane orientation [10]). Large angle differences among sequential normal or binormal vectors can result in inconsistency of resliced planes orientation. This was addressed by rotation of the plane around the tangent to minimize the angle difference.

#### Simplified 3D plane

The initial resliced plane was simplified as a horizontal plane with zero Z axis and normal vector as [0 0 1]. The points in the initial plane were defined as:

$$X_{i,j,k}=\left\{X_{i},X_{j},X_{k}\right\},\text{i}\in \left[1,\;2\text{R}+\text{1}\right],\;\text{j}\in \left[1,\;2\text{C}+1\right],\text{k}=\text{1}$$ (1)

$$X_{i}\in \left[-R\;R\right],\;X_{j}\in \left[-C\;C\right],X_{k}=0.$$ (2)

R and C are the scopes of 2D image grid or width of 2D array and *i*,*j*,*k* are coordinate indices. Matrix *CSA*_1_ was used to describe the initial plane as:

$$CSA_{1}=\left(\begin{array}{lll} X_{-R,-C,1} & \cdots & X_{-R,C,1}\\ \;\;\;\;\vdots & \ddots & \;\;\;\;\vdots \\ X_{R,-C,1} & \cdots & X_{R,C,1} \end{array}\right)$$ (3)

where the elements were 3D points with xyz coordinates defined above. $a1=\underset{X_{\left(0,-C,1\right)}X_{{}_{\left(0,C,1\right)}}}{\to }$ was denoted as additional axis which is a vector from point *X*_(0,−*C*,1)_ to another point *X*_(0,*C*,1)_. In the spherical coordinate system, a vector was identified by two angles, one was azimuth and another was altitude. The two angles {*θ*_1_, *θ*_2_} were determined by azimuth and altitude respectively.

The initial plane was rotated to a vertical plane and then followed by two rotations, while the other plane *CSA*_*i*_ at point *p*_*i*_ was rotated twice over previous planes. Rotation axes were defined as *a*1 and $n_{p_{i}}$, where the rotation matrix *F* was computed by {*θ*_1_, *θ*_2_}, $\left\{a1,n_{p_{i}}\right\}$ followed by Rodrigues' Rotation Formula [13] and *a*1 was updated after the transformation. Trilinear or spline interpolation was used to restore the gray intensity at new coordinates.

In the above procedures, directional axis *a*1 plays the role of a sweeping line in traditional CPR[14], where the NURBS surface can be directly generated by lofting these sweeping lines [15]. This surface construction was equivalent to flattening a curved plane in the traditional CPR.

#### Consistency implementation

Consistency was implemented by rotating axis pairs {normal, binormal} around tangent to make local axes smooth along the skeleton. Since the tangent must be fixed along the curve direction, the resulting pair {normal, binormal} cannot be the same as the previous point because they may have a small angle between two sequential normal vectors when the spatial curve direction changes. The minimization of angle difference was converted into a search for minimal angle difference:

$$\sum_{i=1}^{N-1}\underset{\theta }{\text{max}}\left\{dot(n_{p_{i},}n_{p_{i+1}})|\theta_{p_{i,i+1}}\in [1^{{}^{\circ}},180^{{}^{\circ}}]\right\}$$ (4)

#### Transformation procedures

General space transformation was defined as position mapping ***F***, where the point in original image space was denoted as $\vec{X}$. An example of vector $\vec{X}$ is a 3D pixel on an image grid, or an interpolated point at sub-pixel level given by:

$$\vec{Y}=F(\vec{X})$$ (5)

$\vec{Y}$ was defined as re-sliced or re-sampled data. The image processing ***P*** was regarded as a function imposed on $\vec{Y}$ and $\vec{M}$ was 3D binary segmentation as:

$$\vec{M}=P(\vec{Y})$$ (6)

$$\vec{X\prime }=F^{-1}(\vec{M})$$ (7)

**Fig 5:**
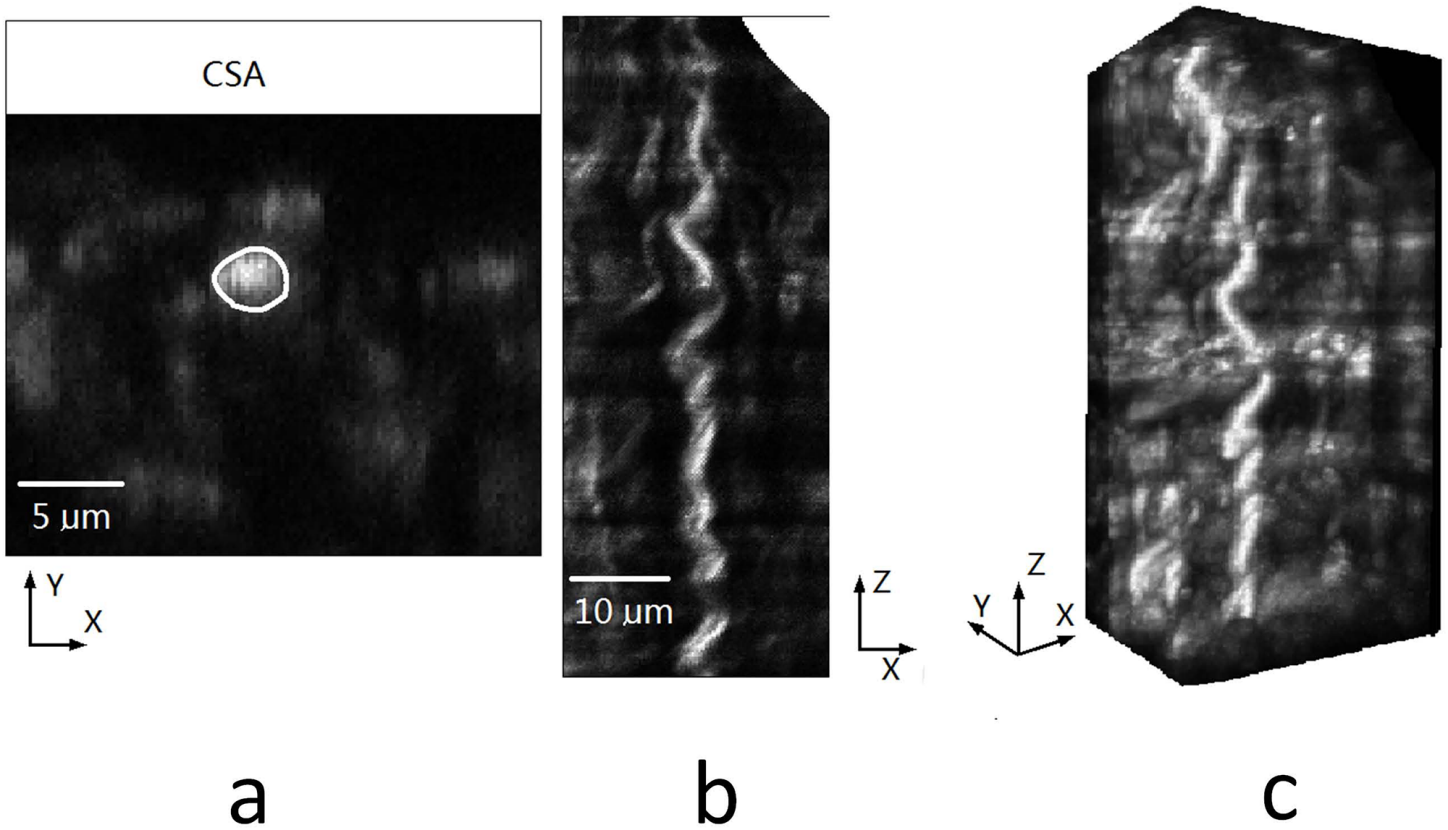
**a)** Cross section plane with a white contour drawn. b) One permuted image slices from another viewpoint. c) The rendering 3D volume of permuted images.

The segmented $\vec{M}$ was mapped back into $\vec{X\prime }$ in original space. ***F*** was an affine transformation that included rotation, translation, or scaling. $\vec{Y}$ was pixel/voxel, but ***F***^**−1**^ may cause $\vec{X\prime }$ to be a point cloud. The geometrical measurement was operated in $\vec{X\prime }$ or $\vec{M}$. The tilt angle, waviness (tortuousity ratio), and orientation were measured in original space, where CSA can be measured on $\vec{Y}$ in resliced image space. The result of CPR is illustrated in Fig 5 including a contour in one cross section plane and a permuted resliced image stack.

### Region segmentation and skeleton

**Fig 6:**
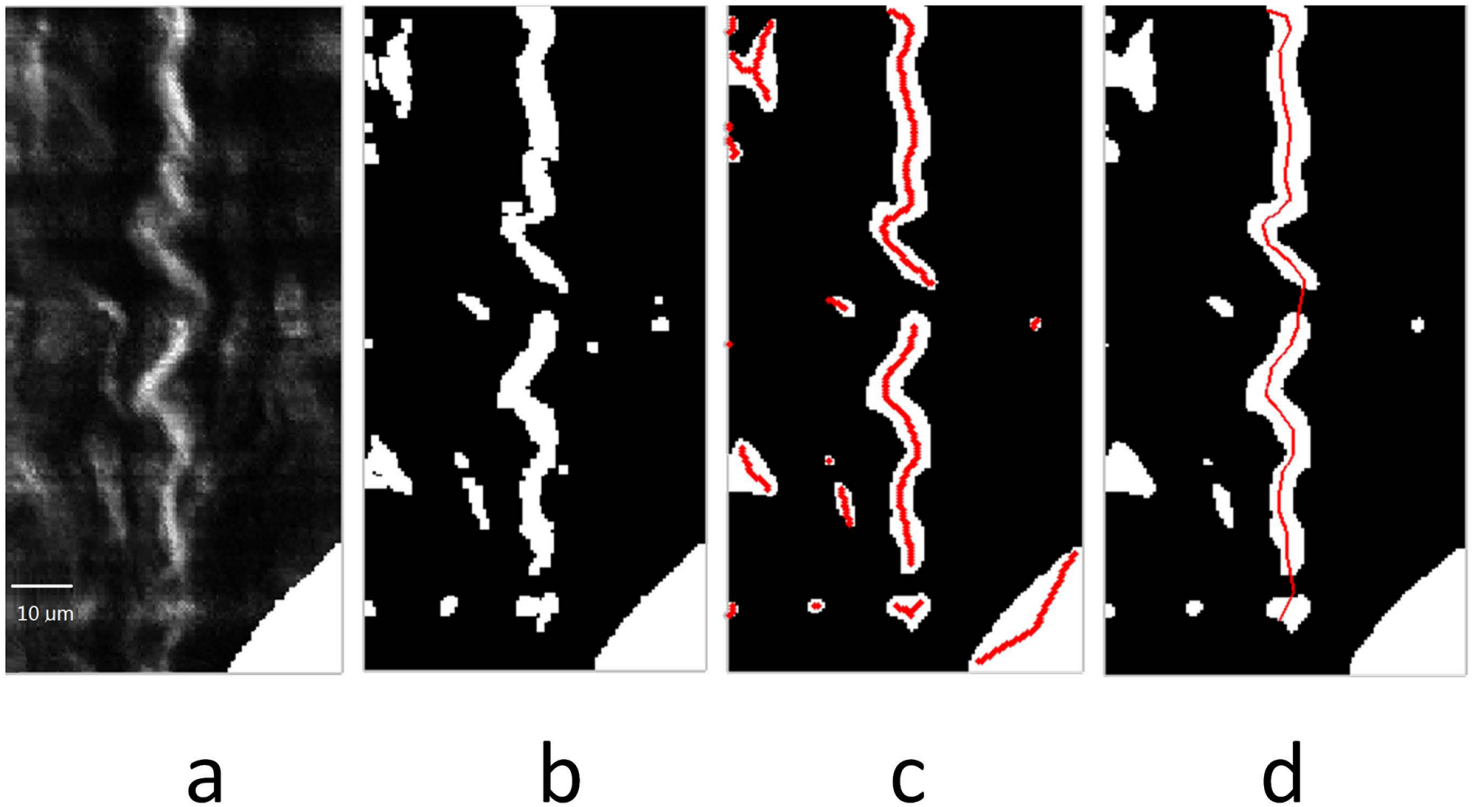
a) Resliced image example. b) Binarization result. c) Smoothed result with overlapped skeleton. d) Refined skeleton of Spline interpolation result for waviness and tilt measurement.

The resliced images were overlapped in 3D where the collagen fiber segmentation was processed by a semi-automatic method in the public ImageJ toolbox, where there are multiple binarization methods including ostu, maxentropy and others. Based on binary results, the skeleton was extracted as an invariant feature such that waviness, tilt angle, CSA, and other specific 3D parameters were obtained without ambiguity. A 2D Skeleton extraction can be simplified in resliced 2D images as shown in Fig 6a. Image processing was implemented in ImageJ and the skeleton obtained in Fig 6c was used for further processing. Gaps in the skeletons were interpolated by spline as in Fig 6d.

**Fig 7:**
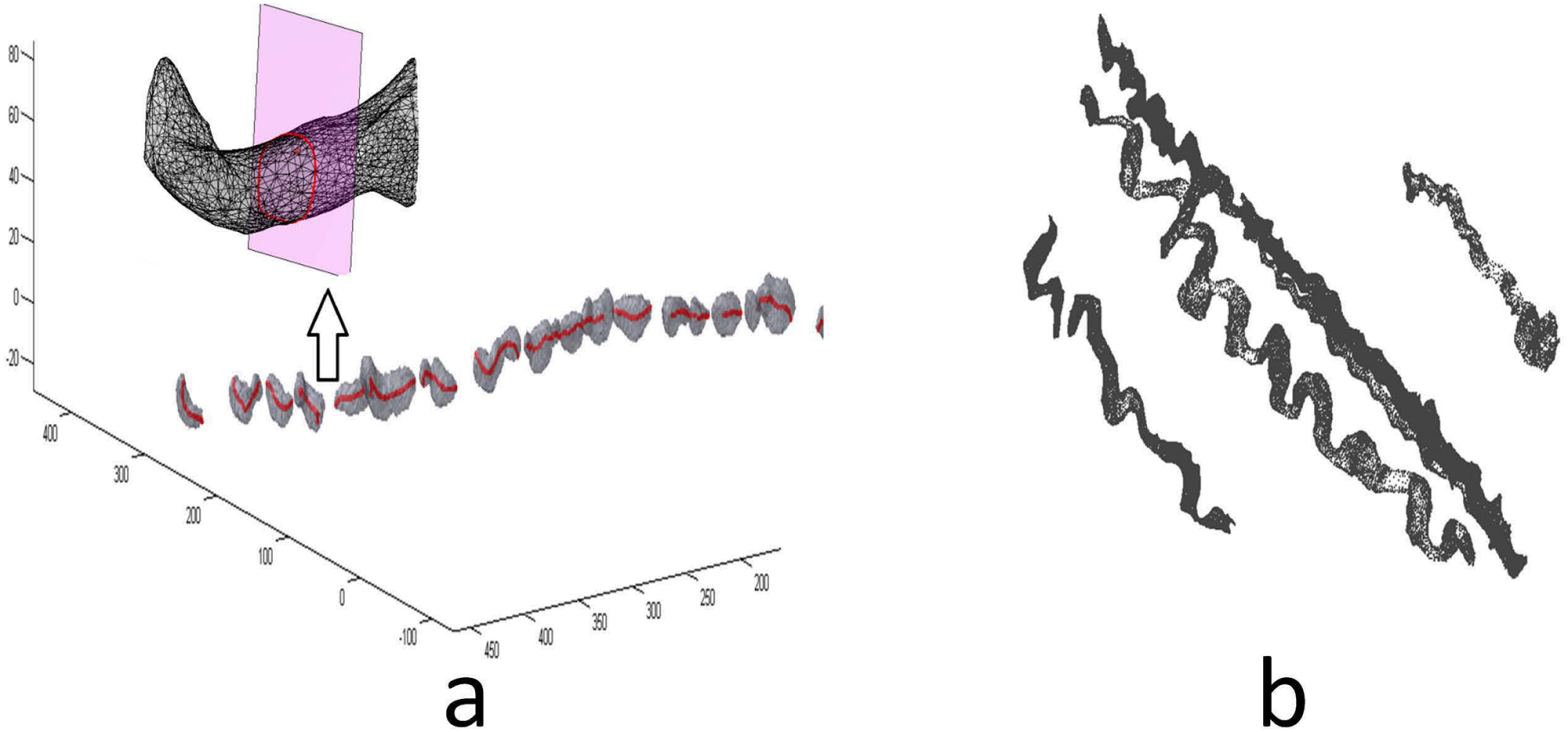
a) 3D surface mesh of segmented collagen fiber. Red was skeleton. Cross section area was illustrated above the mesh. b) Examples of reconstruted 3D collagen fibers from one 3D image stack.

The 3D skeleton was obtained directly from 3D segmented objects at the accuracy level of voxel or sub-voxel, which is dependent on the algorithm used. In this investigation, fast marching [16–18] was used to provide a skeleton with sub-voxel accuracy. The initial ordering of skeleton points was refined by trimming to obtain the main trunk of skeleton. Branches may be caused by artifacts or inaccurate 3D regions. Tree structures with branches were trimmed to leave a main trunk (see S1 Appendix, Algorithms A and B). Algorithm A ordered all skeletons into a coordinate list, while algorithm B removed branches and maintained the main trunk skeleton in each connected region. As the 3D segmentation result contained some gaps, the low intensity region was segmented as gaps or background region. The entire skeleton was restored into one single curve which was completed by spline interpolation after 3D ordering of skeleton points. A 3D fiber reconstruction with its skeleton is illustrated in Fig 7a. Several reconstructed collagen fibers from the same image stack are illustrated by MeshLab in Fig 7b.

### Geometrical parameters

#### Waviness

Waviness *w* was defined as the ratio of curve length *C*_*ij*_ and straight length *l*_*ij*_. The curve length was defined as the distance between each pair of neighboring curve points and the straight line was defined as the Euclidean distance of two end points in the curve. $C_{i}=\left\{C_{i_{x},i_{y},i_{z}}\right\}$ is the definition of a point with coordinates xyz:

$$\begin{array}{lll} w=\frac{C_{ij}}{l_{ij}}, & C_{i,j}=\sum_{k=2}^{k=j}l_{(k-1),k}, & l_{ij}=dist(C_{i},C_{j})={\left\|C_{i}-C_{j}\right\|}_{2} \end{array}$$ (8)

where *k* is the index of point *C*_*k*_, and *i*, *j* are the start and end points. Waviness was computed on 3D skeletons directly or on 2D skeletons extracted from 2D resliced images and restored to original positions. The extracted skeleton by fast marching method was not an ordered curve. The length of skeleton for the entire fiber skeleton was defined as the main trunk.

#### Cross sectional area

The cross section of a fiber was defined as a polygonal plane because it was a closed polygon area intersected with fiber surface mesh. The skeleton tangent vector played the role of intersection plane normal. A circle was approximated to fit this CSA. Although the CSA did not have a perfectly circular shape, the simplification was sufficient for collagen fiber measurement. Fig 7a shows the CSA on a mesh.

Based on the skeleton, the ratio of maximal difference of Z axis coordinates and the straight line distance in XY plane was converted into tilt angle *T* which was also called latitude (radial) angle. The skeleton obtained in resliced image space was converted into real coordinates in the original space as:

$$t=\frac{\text{max}(C_{i_{z}})}{l_{ij}}$$ (9)

#### Orientation

Accurate orientation required large amount of sample data. As a well-studied topic, orientation measurements from either global or local methods have abundant analysis in the literature [19–21]. Moreover, the orientation accuracy was not affected by 2D or 3D structure. The orientation was measured by MatlabRegionProps as described previously [22].

## Results

**Fig 8:**
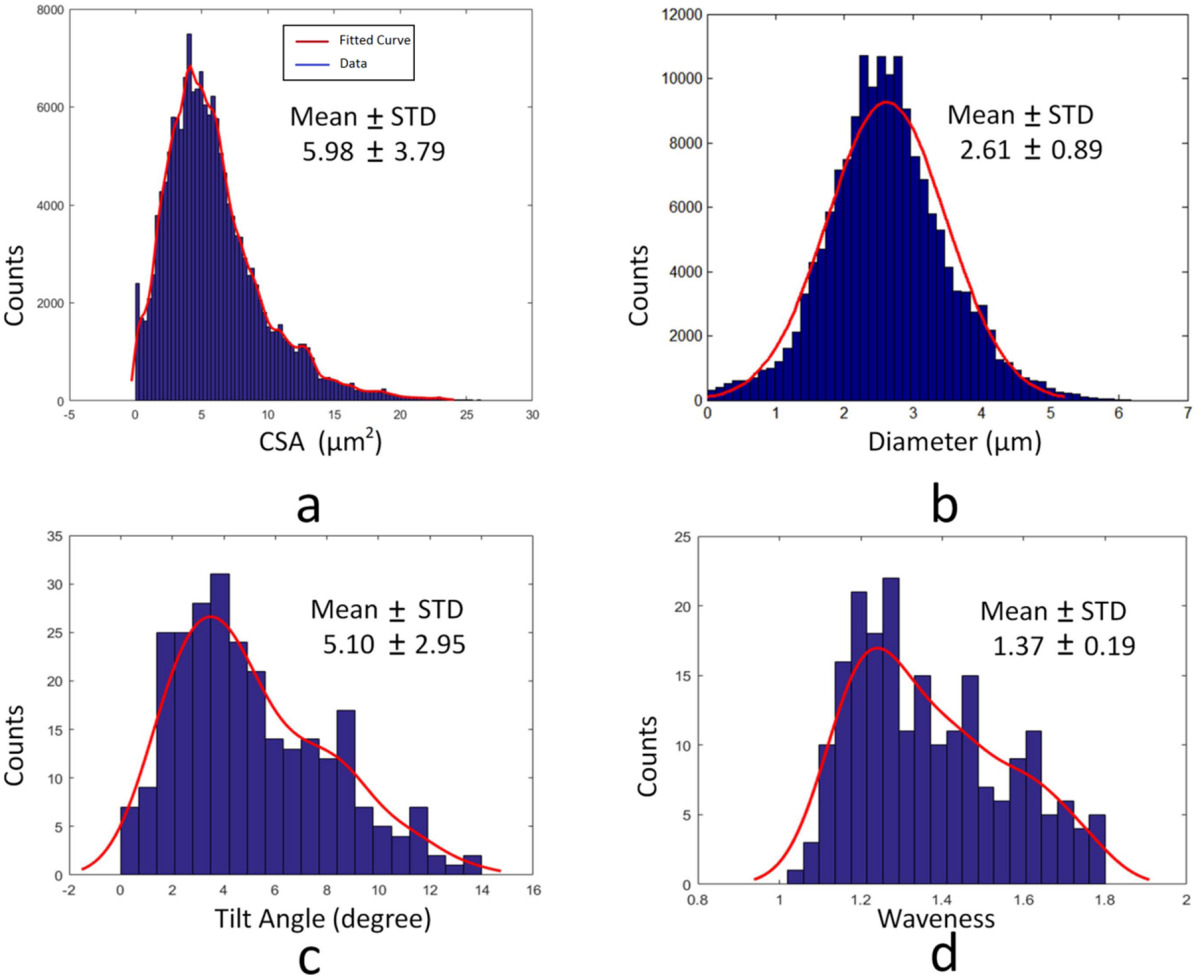
a) CSA result. b) Diameter result. c) Tilt angle result. d) Waviness result.

The CSA and diameter results from over 100,000 measurement points along 80 collagen fibers are illustrated in Fig 8a and 8b, where measurements were taken along the length of the collagen skeleton. Since the normal plane was described mathematically as one normal vector in a 3D plane, it can produce multiple intersected areas with the entire fiber surface mesh. Only the area of the center point near the skeleton was analyzed. Previous manual measurements [11, 12] indicated a width of ~2.8 μm, as compared to current diameter computation of 2.61±0.89 μm with a corresponding CSA of 5.98 ± 3.79 μm^2^. There was not a statistically significant difference between 2D previous manual measurement and current 3D measurement of collagen fiber diameters (p-value = 0.107). Diameter measurements larger than 6 μm or other predefined threshold were regarded as outliers. As the skeleton curve was interpolated by B-spline, the number of new interpolated points was three times more than the original. Hundreds of measurement points in each fiber were sampled for a total of more than 100,000 sample points. The number of outliers was generally < 1% of sample size. The outliers were mainly derived from inaccurate normal vectors and unsmooth fiber surfaces. The center difference was used to compute the normal vector in each curve point.

The tilt angles are illustrated in Fig 8c and found to be 5.10±2.95°. The actual shape of collagen fiber was not always a straight line emitted from one point with a latitude angle, but rather it was possible to have waviness in all three axis of XYZ space. The use of tilt definition was a simple description of Z axis coordinate variation.

**Fig 9:**
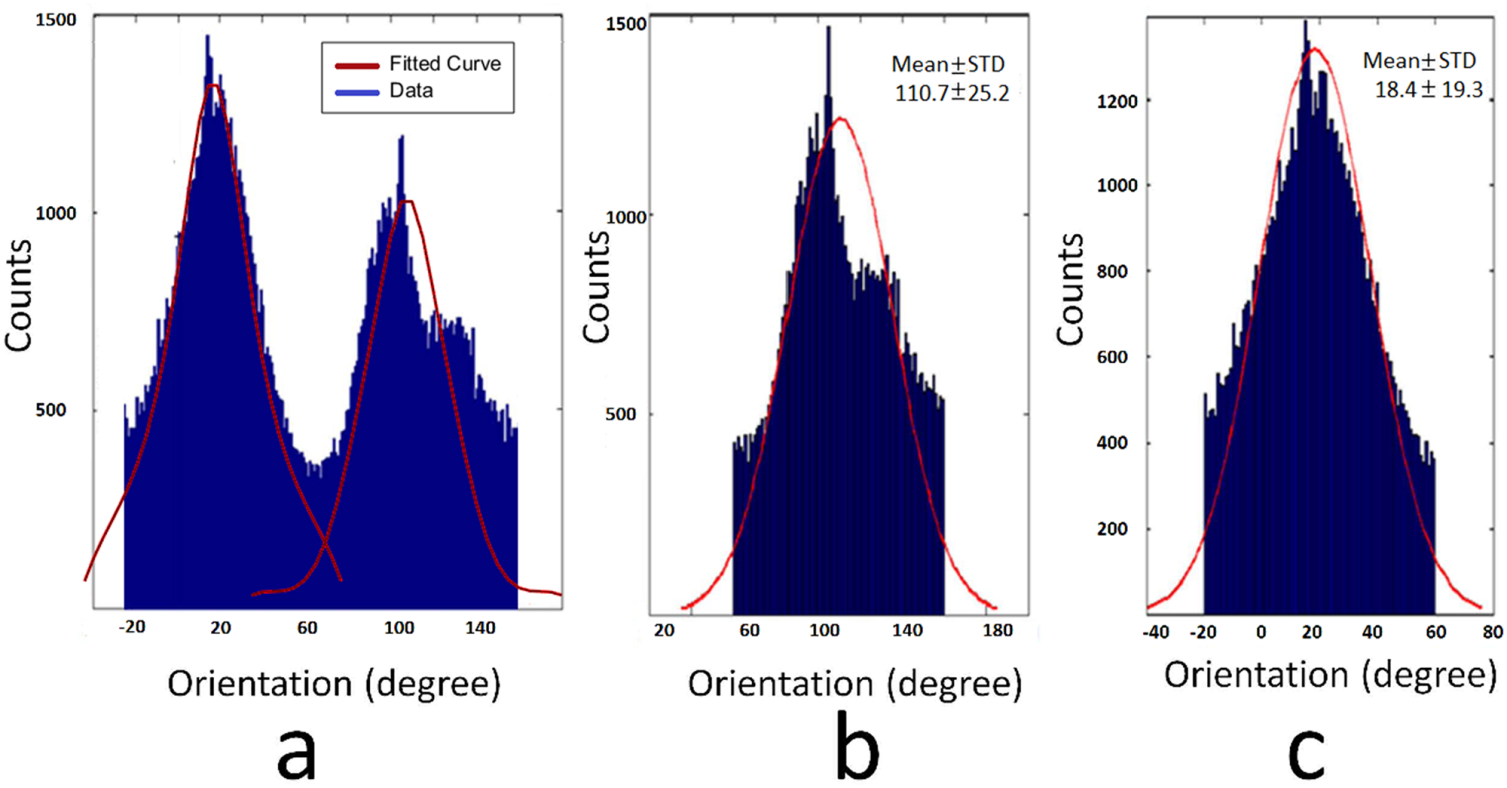
Orientation result. a) Original angles. b) One distribution was extracted with a mean of 110 degrees. c) Another distribution with a mean at 18 degrees.

Fig 8d shows the waviness of collagen fibers. The mean± SD of waviness was 1.37±0.19 in original space, as compared to previous manual measurements of 1.30± 0.11 [11, 12]. The orientation histograms are depicted in Fig 9 and the distributions were similar to previous manual measurements. All direct measurements of orientation are illustrated in Fig 9a, where the angle definition range of 0–180° was based on the coordinate system in Fig 2. The biaxial distributions had two different peaks at 110.7± 25.2 and 18.4± 19.3° in Fig 9b and 9c, respectively. The orientation was computed by MATLAB function, and the negative value around 0° was regarded as belonging to the distribution where the second peak was 18.4°.

**Fig 10:**
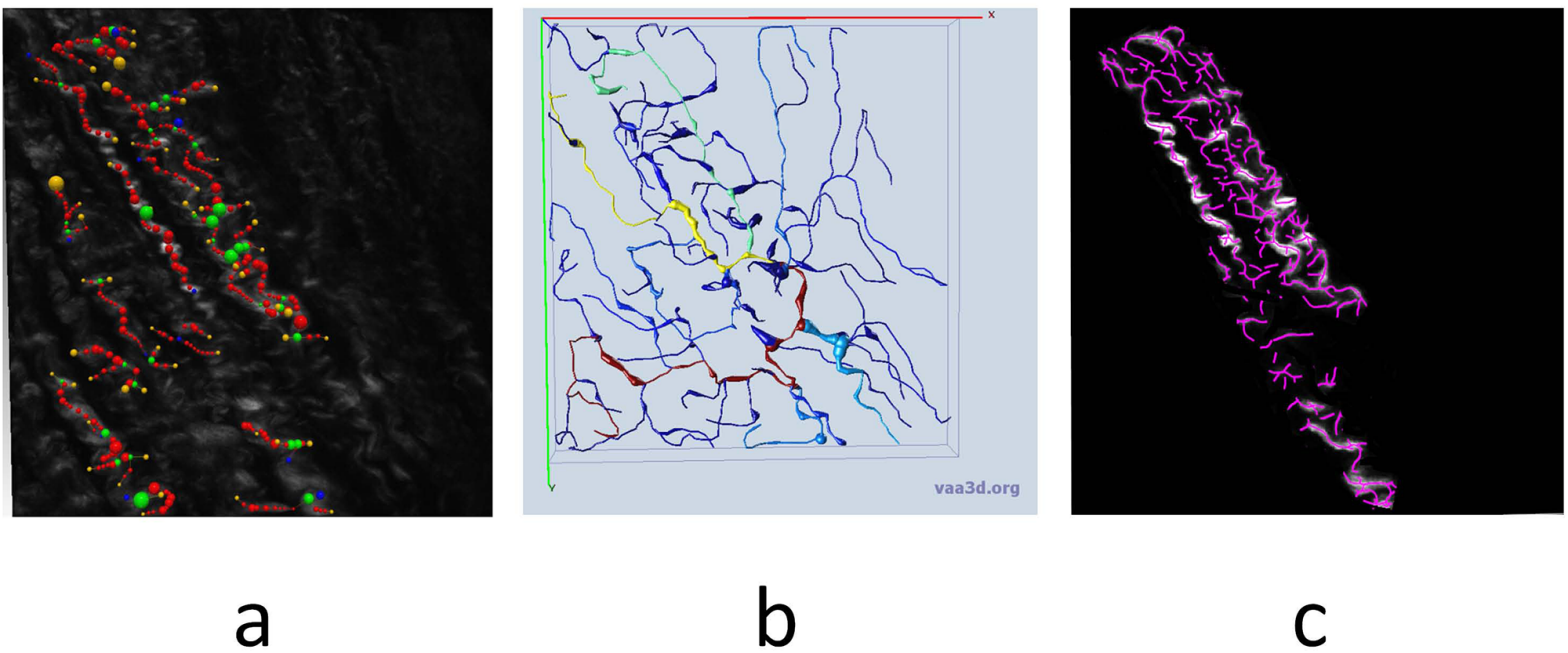
a) NeuTu processing result. b) Vaa3D result. c) SOAX result.

Finally, typical fiber structure was processed by open source software Vaa3D, NeuTu and SOAX as illustrated in Fig 10. The tortuosity was not recognized correctly in some locations while the fuzzy regions were ignored. The high curvature regions were overlooked by NeuTu and Vaa3D in Fig 10a and 10b, and SOAX had more irrelevant branches in Fig 10c.

## Discussion

This is the first study to provide a statistically validated quantitative algorithm for the reconstruction of 3D structure of individual collagen fibers within the coronary arterial wall. The 3D segmentation of single collagen fibers was based on a novel method using arbitrary resliced planar reformation.

Although sophisticated machine learning methods have been employed in the reconstruction of coronary artery collagen fibers in similar images [23], practical algorithms for collagen fibers have not been well investigated. The collagen fibers in our study demonstrated a large variation of geometrical features. In addition to collagen fiber tortuosity, the fuzzy or blurred edges resulted in segment gaps and overlapped regions as observed in MPM images. Our method underscored the new sampling technique which can be followed by a simple region segmentation method. In the resliced image space, the stacked 2D slices displayed clear structure and tortuous features were reduced.

Collagen fibers similar to ours have been analyzed [24, 25] to obtain the angular distribution from whole images, but not individual fibers. Fourier transform used in angular or orientation distribution is a global feature analysis, where an orientation-dependent co-occurrence matrix (OD-GLCM) method was proposed to improve orientation estimation [26]. Wedge filtering and varying length-scale analysis were employed towards a fully automated modeling parameter extraction [19]. Local geometrical parameters, like waviness and orientation, were measured by semi-automation and manual tracing [21], where some 3D measurements were decomposed into 2D measurements. Individual fiber parameters including 3D waviness, 2D diameter, and 3D CSA, however, have been less well investigated because they require local image processing methods and 3D coordinates.

The primary obstacle in the analysis of tortuous collagen fibers lies in the lack of simple and straightforward visualization. Without multiple observation viewpoints from outside and inside of the fiber structure, it is difficult to visually inspect and manually intervene in image segmentation. Some demonstration of visualization is illustrated in Ref. [25], but the effect was restricted to high image quality with advanced imaging techniques in some tissues. The gaps and branching in low quality images remains a critical topic in object recognition.

A typical task in scientific visualization is to cut through volume data. Many open source code platforms provide this functionality. MPR was implemented by simple vtkExtractVOI and sophisticated vtkImageReslice filter, from slicing parallel to the image axes to plane cutting with arbitrary orientation. CPR has also been called 3D curved MPR. In 3D tubular structure analysis, the advantage of CPR is that the cutting plane can be defined in any direction and angle. Since the resliced plane may be constructed along a curve, it is called Curved Planar Reformation. The flattening of a curved plane into a 2D plane can provide a user straightforward visual effect.

One of the aims of this study was to reconstruct the 3D geometry of collagen fibers with a simplified reslicing method which mathematically unifies the MPR and CPR methods. In the resliced image space, segmentation and visualization was performed with enhanced visual confidence. An additional aim was to reconstruct the 3D coordinates of collagen to extract the fiber skeleton as the measurement basis of various morphological parameters.

Since each collagen fiber provides only one tilt angle and waviness, skeletons from over 200 fibers were used to provide tilt and waviness measurement without reconstruction of their surfaces. Since the 3D waviness measurement was based on the 3D skeleton, it was unnecessary to reconstruct the surface mesh. The procedure was demonstrated in Fig 7 where the unrefined skeleton obtained in Fig 7c can be manually adjusted by removing irrelevant portions. As the binarization of fiber and skeleton refinement were made in 2D images, it took about 10 times less time than reconstruction of all 3D coordinates. The measurements of tilt and waviness from 200 fibers had sufficient statistical power.

As compared to previous manual measurements of 2D waviness at 1.30 ± 0.11, the 3D waviness computed in this study was 1.37±0.19. The agreement is excellent given that manual 2D measurement was based on manual tracing, which tends to localize segments at high intensity regions, whereas 3D skeleton or ridge detection may contain some low intensity regions. For our new analysis of orientation, the differences from previous manual measurements of 110±28.0 and 22.7±12.6° were small [11, 12].

From the perspective of computerized algorithm, a 3D skeleton is the most difficult topic in feature extraction. In the selection of voxel-based and sub-voxel based methods, a 3D thinning algorithm [27, 28] is not used routinely because of a low level of accuracy. The branch points of skeletons created by a voxel-based method were relatively easy to recognize. In the sub-voxel methods, fast marching [16,18,29] and mesh contraction are typical methods used. Fast marching was used in this study since mesh contraction can introduce small branch artifacts. The sensitivity was partially caused by the coarseness of 3D segmentation results.

The 3D segmentation results were refined iteratively in both image grid and mesh domains. It is impossible to extract smooth and accurate binary objects at the initial steps. The initial regions were considered as both under- and over-segmentation results which contained noise and irrelevant regions. Adhesive branches from the fiber itself and other fiber segments were also included. In order to overcome the over-segmentation, there were two post-processing targets (gaps between segments and branches of skeleton). Since the single fiber was the focus of this investigation, the irrelevant segments or overlapped branches were removed with a semi-automated method. Region growing was employed by restricting the computation to a user-defined local region, and other techniques (e.g., level set methods) were used by trial and error. Irrelevant regions were also removed by mesh smoothing and deletion. To improve computational efficiency, it was important to include manual selection of a region of interest as well as user-drawn initial skeletons close to the fiber ridges. The algorithm test code can be available at https://figshare.com/authors/Tong_Luo/780424.

## Limitations

The collagen fibers were processed in a limited quantity, as the imaging quality degraded at the high curvature segments and branch positions. The image feature therefore demonstrated low intensity or background, weak boundary (gradual variation intensity), and entangled segments exacerbated recognition of tortuous collagen fiber. Although CPR was used to browse structure with more viewpoints, the branches or overlapping problem required manual intervention. The collagen fiber network structure pattern has not been analyzed and categorized, and there is currently no standard data for image feature extraction validation. It is necessary to obtain more accurate geometrical parameters through a larger scale collagen fiber database, and to further improve algorithm efficiency.

## Summary

Collagen fibers were analyzed by arbitrary resliced planar reformation. The simplified visualization method can be applied to the imaging of various fibrous tissues. To overcome the inaccuracy of segmentation results, flexible mesh smoothing and deletion in the graph domain improved the manual intervention efficiency. The skeleton was emphasized with a new refinement algorithm for measurement of geometrical parameters. This novel reconstruction approach provides a detailed database on the collagen fibers to serve as a basis for micro-structural mechanical models of the coronary vessel wall.

## Supporting information

S1 Appendix Algorithms for skeleton ordering and branch triming. (DOCX)
