# Supplementary material for: Resliced image space construction for coronary artery collagen fibers
Source: PLoS One. 2017 Sep 27;12(9):e0184972. doi: 10.1371/journal.pone.0184972 (PMC5617181; doi:10.1371/journal.pone.0184972)
Supplement: S1 Appendix — (DOCX) [file pone.0184972.s001.docx]

**Appendix**

**Algorithm A. Skeleton Ordering**

# Input: multiple segments of 3D curves.

# n is the number connected regions, S1 is skeleton, S1=S10 S11 means the ordering from start to end point.

# Voxelisation of each separated Surface mesh.

# 3D skeleton by fast marching for each region.

Output: ordered skeleton segments.

# For i=1 <-- to n do

# While in each mesh

**Distance(***S_1,_ S_2_***) 🡨 min ( distance (** *S_1i ,_ S_1j_***)),**

# DistMap(i,j) 🡨 Distance(*S_i,_ S_j_*)

# Start skeleton 🡨 any *S_i_*

# *S_j_* 🡨 argmin_j_ (DistMap(i,:))

*S_k_* **🡨***S_mi_ S_nj_* **🡨** argmin_ij_ ( length(*S_mi ,_ S_nj_*))

# compare curve length in all linking combination

# {a0-a1-b0-b1, a0-a1-b1-b0, a1-a0-b0-b1, a1-a0-b1-b0 }

# find minimal length

# ordering two segments and merge into one segment

# set as next start segment

# end

end

**Algorithm B. Branch Triming**

# Input: multiple segments of 3D curves.

# Output:

# While in each mesh

#

# Branches point *Bi*🡨{ *i*|Number(DistMap(*i*,:)<*T*)>=3}

# While ~isempty(*S_all)_*

# New merged *Sk*🡨 minj(Distance(*S_i,_ S_j_*))

# remove other *B_i_* , *S_i,_ S_j_* in *S_all_*

end

# main trunk *S*🡨*Sk*
